# Fine-Tuning Large Language Models for Effective Nutrition Support in Residential Aged Care: A Domain Expertise Approach

**DOI:** 10.3390/healthcare13202614

**Published:** 2025-10-17

**Authors:** Mohammad Alkhalaf, Dinithi Vithanage, Jun Shen, Hui Chen (Rita) Chang, Chao Deng, Ping Yu

**Affiliations:** 1 School of Computer Science, Qassim University, Qassim 51452, Saudi Arabia; mohklf@gmail.com; 2 Centre for Digital Transformation, School of Computing and Information Technology, Faculty of Engineering and Information Sciences, University of Wollongong, Wollongong, NSW 2522, Australia; dsv912@uowmail.edu.au (D.V.); jshen@uow.edu.au (J.S.); 3 School of Nursing and Midwifery, Western Sydney University, Penrith, NSW 2751, Australia; rita.chang@westernsydney.edu.au; 4 School of Medical, Indigenous and Health Sciences, University of Wollongong, Wollongong, NSW 2522, Australia; chao@uow.edu.au

**Keywords:** large language model, domain-specific fine-tuning, RoBERTa, prediction, nursing notes, unstructured EHR, malnutrition

## Abstract

Background: Malnutrition is a serious health concern among older adults in residential aged care (RAC), and timely identification is critical for effective intervention. Recent advancements in transformer-based large language models (LLMs), such as RoBERTa, provide context-aware embeddings that improve predictive performance in clinical tasks. Fine-tuning these models on domain-specific corpora, like nursing progress notes, can further enhance their applicability in healthcare. Methodology: We developed a RAC domain-specific LLM by training RoBERTa on 500,000 nursing progress notes from RAC electronic health records (EHRs). The model’s embeddings were used for two downstream tasks: malnutrition note identification and malnutrition prediction. Long sequences were truncated and processed in segments of up to 1536 tokens to fit RoBERTa’s 512-token input limit. Performance was compared against Bag of Words, GloVe, baseline RoBERTa, BlueBERT, ClinicalBERT, BioClinicalBERT, and PubMed models. Results: Using 5-fold cross-validation, the RAC domain-specific LLM outperformed other models. For malnutrition note identification, it achieved an F1-score of 0.966, and for malnutrition prediction, it achieved an F1-score of 0.687. Conclusions: This approach demonstrates the feasibility of developing specialised LLMs for identifying and predicting malnutrition among older adults in RAC. Future work includes further optimisation of prediction performance and integration with clinical workflows to support early intervention.

## 1. Introduction

Malnutrition is a serious health problem with many negative health consequences for older people, such as a weakened immune system and impaired cognition [1]. It may also contribute to vulnerabilities to infections, anaemia, and other diseases [2,3,4]. Malnutrition has been identified as a key area for urgent review by the Australian government for residential aged care [5] with identification of poor nutrition and weight loss as essential indicators measuring the quality of care in residential aged care facilities (RACFs). Healthcare professionals are requested to screen older adults for early detection of malnutrition regularly [6,7]. To date, the common malnutrition screening tools used at RACFs include Mini Nutritional Assessment (MNA) and Subjective Global Assessment (SGA). However, since using these tools is time-consuming, they are not consistently applied [7]. Predicting and addressing malnutrition can lead to better health outcomes and improved quality of life [8]. Thus, it is crucial to develop new methods to improve the efficiency and effectiveness of malnutrition detection. However, the scarcity of reliable datasets detailing nutritional intake, the lack of domain-specific knowledge models, and the novelty of the transformer approach have been primary obstacles to developing a malnutrition prediction model for older people.

### 1.1. Electronic Health Records

Electronic health records (EHRs) have been widely adopted in RACFs in Australia to document clients’ diagnosis, health assessment, nursing care plans, personal preferences, activities of daily living, and care received [9]. The datasets in these EHRs can be classified as structured data and unstructured data. The structured data include client demographics and diagnoses that are recorded in structured tables. The unstructured data include nursing care plans, assessment records, and free-text clinical notes [10]. Most information about clients in RACFs, including nutritional information, is recorded in unstructured progress notes in the EHR. Since EHR data are captured in real time in the care service delivery process, models trained on EHR can be more readily applied to clinical practice [11]. This provides an opportunity for natural language processing (NLP) to extract insights from unstructured data in EHRs for aged care services.

### 1.2. Natural Language Processing

Recent advancements in artificial intelligence, more specifically NLP, have opened doors for extracting relevant information and automating clinical diagnoses and predictions using language models on patient EHR [12,13,14]. One of NLP’s recent advancements is the word embedding technique, which represents text as multi-dimensional vectors. Each word in a corpus is mapped to a specific vector, represented by numeric values. These values are learned after training the model on a large corpus. After training, the model can understand raw text because similar words will have similar representations. Models such as GloVe [15] and word2vec [16] apply such text representation and have achieved promising results in different fields [17,18]. However, these models lack context awareness, a core competency in text analysis.

### 1.3. Large Language Models

The emergence of encoder-based large language models (LLMs) such as BERT [19] and RoBERTa [20], has brought positive disruption to the field of NLP. They utilise contextualised embeddings that account for both the prior and subsequent contexts of a token, adjusting its weight vector accordingly. By analysing relationships between all pairs of words, LLMs introduce context awareness, addressing the weakness of GloVe and word2vec models. LLMs can also transfer previously acquired knowledge, making them more efficient and achieving state-of-the-art (SOTA) performance in many general downstream tasks with minimal to no need for architectural modification [19]. They can be further pre-trained on a specific corpus for domain-specific tasks. This has enabled them to be successfully fine-tuned for various complex applications. Previous studies have demonstrated that LLMs can be trained on medical corpora to achieve high reliability in medical diagnoses and predictions [20,21,22]. While LLMs have shown their utility in extracting data from public health datasets, their practical application in specific clinical tasks within real clinical settings, using clinic data, remains limited [23,24].

### 1.4. RoBERTa

RoBERTa is a robust encoder-based LLM that is further optimised from its predecessor, the BERT model, for better performance on a variety of NLP tasks [25]. It has achieved SOTA performance after being trained with massive text data with increased parameters, larger batch size, and learning rate. RoBERTa utilises byte-level tokenisation instead of the word-level tokenisation in BERT. In addition, it randomises the masking position, which eliminates the chance for the model to memorise the training data. Previous studies found that encoder-based LLMs such as RoBERTa outperform or at least are as effective as decoder-based LLMs, e.g., ChatGPT, in classification tasks [26,27]. RoBERTa’s architecture is highly suitable for fine-tuning on domain-specific datasets. It has a smaller model size, requires less computational power and memory, and often provides faster inference times compared to larger models like the Llama model [28] or ChatGPT [29]. These make it more feasible for deployment in various health systems and devices [30]. Therefore, we choose RoBERTa as the candidate model for our task of generating knowledge about nutrition care in RACFs over other models.

### 1.5. Objective

Since, to date, there are no models that have been reported to be fine-tuned explicitly for classifying and predicting malnutrition in older people, this study aimed to conduct NLP on free-text notes in the RAC EHR for two downstream tasks: (1) identifying malnutrition notes and (2) malnutrition prediction. We fine-tuned the RoBERTa encoder-based language model to produce a nutrition domain-specific language model in the Australian RAC setting. We evaluated the performance of our model in comparison with other baseline models, including Bag of Words, GloVe, BioClinicalBERT, and RoBERTa. Free-text nursing notes within EHRs often contain extensive and detailed documentation; however, RoBERTa has a maximum sequence length limitation of only 512 tokens. To address this challenge, we developed a new method for processing long notes with lengths exceeding the 512-token limit.

Overall, the contributions of this study include: (1) fine-tuning a domain-adapted LLM for malnutrition prediction, (2) integrating the domain-adapted LLM to enhance clinical relevance and improve information retrieval and predictive performance from free-text EHR data, and (3) developing a method to handle long nursing notes by truncating and aggregating text into segments that fit within RoBERTa’s 512-token limit.

### 1.6. Organisation of the Paper

The remainder of this paper is organised as follows. Section 2 presents the proposed methodology. Section 3 describes the experimental results. Section 4 provides the discussion, including study limitations and directions for future work. Finally, Section 5 concludes the paper.

## 2. Methodology

### 2.1. Dataset

The dataset was obtained from 40 aged care facilities in the state of New South Wales (NSW), Australia. Overall, 4405 de-identified clients’ data were included in this analysis. The data were extracted from 1,616,820 notes of dietitians and nursing care staff recorded between January 2019 and October 2020, with an average number of 366 notes for each client (see Table 1). The human research ethics approval for this study was granted by the Human Research Ethics Committee, the University of Wollongong, and the Illawarra Shoalhaven Local Health District (year 2020).

### 2.2. Data Cleaning

All notes were cleansed of noise, including removing white spaces, special symbols, and unwanted characters that do not contribute to the meaning of the text.

### 2.3. Overview of the Methodology

**Figure 1:**
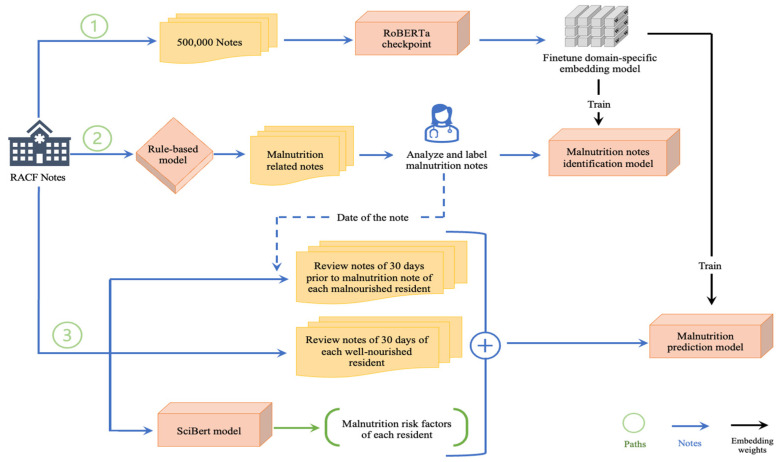
An overview of the model development pathway. Path 1: Pre-training a RAC domain-specific model; Path 2: Fine-tuning a malnutrition note identification model; Path 3: Fine-tuning a malnutrition prediction model.

Figure 1 depicts our NLP pipeline. It consists of three pathways: Path 1, pre-training a domain-specific LLM; Path 2, fine-tuning a malnutrition note identification model; Path 3, fine-tuning a malnutrition prediction model.

### 2.4. Path 1: Pre-Training Domain-Specific Embedding Model

#### 2.4.1. Dataset Construction

We randomly selected 500,000 free-text nursing notes with an average token length of 64. The training dataset included 21,969,925 words, which we considered adequate for domain-specific pre-training, as more notes do not necessarily lead to better results [31]. We then extracted the raw text to a single text file and processed it into chunks of 512 tokens. This chunking procedure resulted in a training set containing 62,273 text chunks (rows).

#### 2.4.2. Model Pre-Training

The weights of the pre-trained baseline RoBERTa model checkpoint (“roberta-base”) were downloaded from the Huggingface transformer library [32]. It contains substantial information regarding the English language corpus. This substantially reduces the pre-training time required to adapt RoBERTa to our specific task compared to training a model entirely from scratch. However, the corpus of nursing progress notes contains many RAC domain-specific terms, abbreviations, and unconventional expressions that are not present in general English, which could affect the performance of the baseline RoBERTa model. Therefore, we chose to train a RAC domain-specific model initialised from RoBERTa on our nursing note dataset to improve the model’s ability to understand the words and phrases used in the RAC nursing corpus. The task for the model is to predict words randomly masked out of an input chunk. The knowledge gained from the resulting model can be transferred and further refined with an additional output layer to create models for various downstream tasks.

Tokenisation was conducted on the nursing text corpus using a pre-trained byte-level tokeniser to fit with the RoBERTa model. We randomly split the dataset into 80% training and 20% validation sets. Then, we set the masking probability to 15% of the words in each input sequence, like the original RoBERTa training. We used whole-word masking instead of token masking for better results [33] (Supplementary Table S1). Additionally, we randomised the masking with each batch to prevent over-memorisation. After that, we trained the model using the following hyperparameters: a learning rate of 1 × 10^−4^, a batch size of 32, and a weight decay of 0.01. The model was trained until the validation loss began to converge, at around 80,000 steps (Supplementary Figure S1). The embeddings of this model were then utilised for the two downstream tasks: malnutrition note identification and malnutrition prediction.

### 2.5. Path 2: Downstream Task 1: Fine-Tuning a Malnutrition Note Identification Model

#### 2.5.1. Dataset Construction

The labelled dataset for malnutrition was based on the work of Alkhalaf et al. [34]. This contained 2278 notes reporting malnutrition (labelled: 1) and 15,000 notes with normal nutrition status (labelled: 0).

#### 2.5.2. Model Fine-Tuning

We further fine-tuned our model with the embeddings from the RAC domain-specific LLM that we built in Path 1 to identify notes related to malnutrition (see Figure 1). We divided the dataset into 85% training and validation datasets, and 15% for the testing set. The hyperparameters included a learning rate of 3 × 10^−5^, a batch size of 16, a weight decay of 0.01, and a 50% dropout rate. We used binary cross-entropy loss with positive weights and the mean pooling output of the last hidden state.

### 2.6. Path 3: Downstream Task 2: Fine-Tuning a Malnutrition Prediction Model

#### 2.6.1. Dataset Organisation

The dataset for this task consisted of the original weekly nursing review notes and the malnutrition risk factors extracted from these notes. Since malnutrition is a health condition that develops over time, to capture each client’s health changes over time, we approached this task as a time series data analysis by extracting the weekly review notes of each malnourished client recorded in the 30 days before the onset of malnutrition. We organised each client’s notes chronologically, with the earliest note appearing first in the sequence and the most recent note appearing last. We followed the same procedures to organise data for clients without malnutrition.

In addition to text-based notes, we also extracted malnutrition risk factors for each client from the notes using the SciBert model for NER with the UMLS linker. In our previous study, we identified 46 malnutrition risk factors in each client’s notes [34,35]. A negation detection technique was applied to distinguish whether a factor mentioned in a note was confirmed or negated [34]. For example, the sentence “no sign of cancer” would be correctly identified as a negation and not counted as a confirmed factor. Each client’s notes and risk factors were then combined into a single dataset, with the notes included as raw text and the risk factors represented as a one-hot encoding tensor (‘0’ for absence, ‘1’ for presence). This combined dataset was subsequently used for the malnutrition prediction model.

#### 2.6.2. Model Fine-Tuning

Our model was initialised from the RAC domain-specific model pre-trained in Path 1. The training dataset consisted of 862 aggregated notes (rows) of malnourished clients and 2298 aggregated notes (rows) of well-nourished clients. We split the data into 85% for training and validation, and 15% for holdout testing. Hyperparameters included a learning rate of 3 × 10^−5^, a batch size of 16, a weight decay of 0.01, and a 50% dropout rate. We used binary cross-entropy loss with positive weights. We concatenated the output with the structured data (malnutrition risk factors). We added a fully connected layer to the concatenated data, with a Sigmoid activation function applied to obtain the final output (Supplementary Figure S2).

#### 2.6.3. Addressing the 512 Maximum Length Challenge

In this downstream task, as opposed to Task 1, the notes were longer due to the inclusion of information gathered over a four-week period. Therefore, we encountered long notes spanning a four-week duration, with an average 644 token length (95% confidence interval: 627.17–663.31). Therefore, the length of certain records exceeded the maximum sequence length accepted by RoBERTa and BERT, which is 512 tokens.

For these long records, we truncated and padded the text sequence into equal 512 token parts. Each part starts with a start sequence token and concludes with an end sequence token. A padding token was added if the last part has less than 512 tokens. Attention masks were also manually added as (1), informing the model to pay attention to the token, or (0), suggesting the model ignore the token.

**Figure 2:**
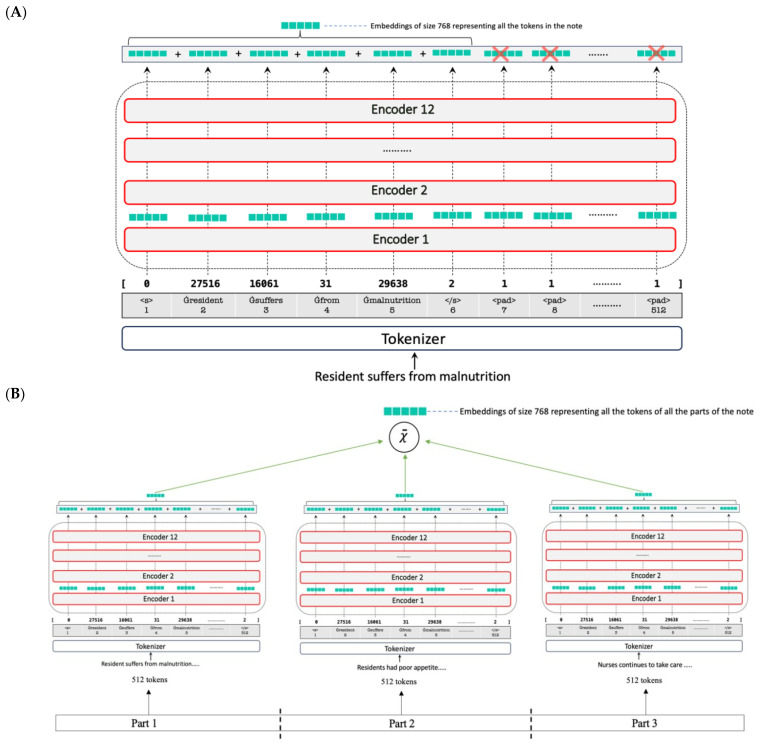
Methods developed for processing long notes with more than 512 tokens. (**A**) Example of a nursing note with sequence length less than 512 tokens; (**B**) Example of a nursing note with sequence length of 1536 tokens. This note is truncated into 3 parts each with a sequence of 512 tokens.

In the model forward function, the last hidden state embeddings of each token, generated by the model, are selectively emphasised through an attention mask. Then, the sum of the masked embeddings is calculated. Only tokens with an attention mask value of 1 are considered; tokens with an attention mask of 0, which indicated a padding token, are ignored. In addition, the model keeps track of the number of tokens. To capture the main ideas of a note, for short notes, embeddings are averaged across all tokens (Figure 2A). Conversely, as longer notes are divided into several parts with an equal length of 512 tokens, embedding tokens are aggregated across all parts (Figure 2B).

### 2.7. Downstream Task Evaluation

All tasks were evaluated using precision, recall, F1-score, specificity, the area under the precision–recall curve (AUPRC), and the area under the receiver operating characteristic curve (AUROC). To better assess the model’s robustness and generalisability, and avoid fine-tuning instability [36], we performed 5-fold cross-validation in each downstream task. We kept the number of epochs in each fold to a low number (four) to avoid the possibility of overfitting the data. In each fold, the model with the least validation loss was utilised for testing on the test dataset. We calculated the cross-validation performance by averaging the k performance estimates of all measures (F1-score, recall, etc.) obtained from the testing sets using the arithmetic mean. Additionally, we calculated the confidence interval for each measure.

This study was implemented using Python 3.10.11, PyTorch 2.0.1, transformers 4.29.2 (Huggingface), and Scikit-learn 1.2.2. Our models were trained on the NVIDIA Tesla T4-16GB graphics processing unit (GPU). 

### 2.8. Statistical Analysis

As the measurement indicators, including precision, recall, F1-score, specificity, the area under the precision–recall curve (AUPRC), and the area under the receiver operating characteristic curve (AUROC), were not normally distributed, we utilised the Mann–Whitney U test. A significant difference is decided if the *p*-value is smaller than 0.05.

## 3. Result

**Figure 3:**
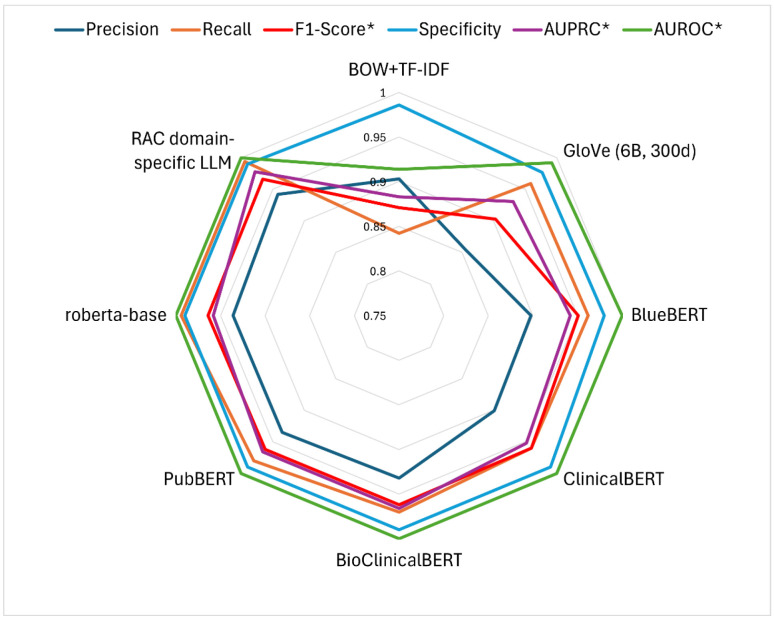
Performance of the five machine learning models on the task of identifying malnutrition notes. F1-score* computed using 0.5 threshold; AUPRC* and AUCROC* computed across various threshold values.

We compared the results of our domain-specific LLM to the other baseline models, Bag of Words (BOW), GloVe embedding, BlueBERT, ClinicalBERT, BioClinicalBERT, PubBERT, and RoBERTa. In the first downstream task, i.e., malnutrition note identification, there were no statistically significant differences in accuracy, precision, recall, or F1-score (Figure 3, *p* > 0.05). Among the models, the RAC domain-specific LLM achieved the highest F1-score of 0.966, closely followed by RoBERTa (F1 = 0.964) and BioClinicalBERT (F1 = 0.962). In contrast, the GloVe model obtained an F1-score of 0.903, while the BOW model recorded the lowest performance with an F1-score of 0.871 (see Supplementary Table S2).

**Figure 4:**
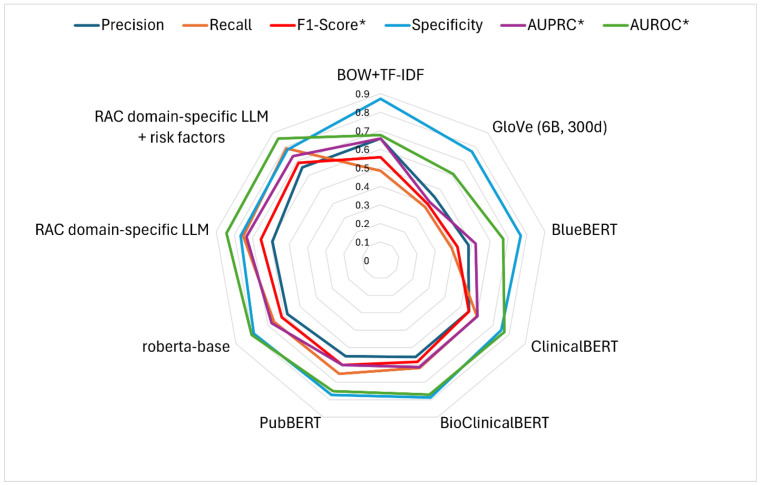
Performance of the five machine learning models on the task of malnutrition prediction. F1-score* computed using 0.5 threshold; AUPRC* and AUCROC* computed across various threshold values.

In the second downstream task, i.e., malnutrition prediction, there were no statistically significant differences in accuracy, precision, recall, or F1-score (Figure 4, *p* > 0.05). Among the models, the RAC domain-specific LLM with the risk factor layer was the top-performing model, achieving an F1-score of 0.687. It was followed by our domain-specific LLM without the risk factors, which achieved an F1-score of 0.655. Next, RoBERTa recorded an F1-score of 0.614, followed by BioClinicalBERT with 0.582. Finally, the BOW model achieved an F1-score of 0.557, while the GloVe model obtained the lowest performance with an F1-score of 0.396 (see Supplementary Table S3). Supplementary Figures S3–S9 present the area under the curve (AUC) plots for each model.

## 4. Discussion

This study aimed to develop an encoder-based RAC domain-specific LLM to accurately identify clients with malnutrition in EHR and create a model capable of predicting malnutrition in older people one month before its onset. We first utilised our RAC domain-specific LLM, initialised by the well-known RoBERTa model and further pre-trained on nursing progress notes. Afterwards, we employed the embedding weights generated from the proposed model for two subsequent downstream tasks. We compared the performance of different parameters on five models: BOW, GloVe, BioClinicalBERT, roberta-base, and our domain-specific LLM. The results illustrated the higher performance of LLMs over BOW and GloVe models. It also demonstrated the advantage of utilising domain-specific embeddings. It is worth noting that this is the first study to utilise LLMs on free-text nursing notes to predict malnutrition in older people, although it has been a health risk that has long plagued the care staff members and has hurt care quality [1]. This study has also developed a method for processing long notes (>512 tokens), which is crucial and particularly relevant in health contexts where it is typical to encounter long and detailed documentation.

For the first downstream task, there have been very few attempts to identify malnutrition notes in EHR in the literature. One attempt to classify malnutrition notes applies the conditional random fields technique to nursing notes [37,38]. However, the study reported that the model performed poorly on malnutrition and had a low F1-score of 0.39. The authors of the study stated that classifying malnutrition notes was very challenging which led to the low accuracy of their model. Another attempt is our previous work to classify the malnutrition notes using a rule-based model which achieved a high-level performance; however, the development of the rule-based method was time-consuming and labour-intensive [35]. To address this limitation, we adopted a domain-specific LLM in this study. The process of fine-tuning and evaluating the model was much more efficient than the rule-based method.

In accordance with the previous reports [20,39,40], LLMs significantly outperformed other comparative models with our RAC domain-specific LLM achieving an F1-score of 0.966, while GloVe and BOW had an F1-score of 0.903 and 0.871, respectively. However, all LLMs yielded comparable performance in this task. We argue that this can be attributed to the notable difference in notes between the positively labelled and negatively labelled instances in the dataset compared to those in the second task.

For the second downstream task of malnutrition prediction, to our knowledge, this is the first study to predict malnutrition in older people in RACFs by applying a transfer learning approach to EHR. LLMs achieved a significantly higher F1-score than BOW and GloVe models. Once again, our RAC domain-specific LLM notably outperformed other LLMs, achieving the highest F1-score of 0.655. This illustrates the importance of pre-training a foundational LLM on a domain-specific corpus. In addition, combining a layer of structured data with the output of the note-based model increased the performance of the model, as evidenced by an increased F1-score (0.687). Despite this, the model did not achieve a high F1-score, unlike in the first task, which is arguably because malnutrition is a health risk influenced by various factors, many of which are prevalent in all clients and are not specific to clients with malnutrition. Therefore, accurately predicting malnutrition is still a complex and challenging mission [6,41]. In future work, we plan to explore advanced techniques such as graph-based feature modelling and attention-enhanced architectures to better capture relationships between clinical features and improve predictive accuracy [41].

For both tasks, the RAC domain-specific LLM did not achieve statistically significant performance improvements compared to other models; however, it consistently outperformed them in all the performance metrics. These results underscore the potential of domain-adapted LLMs to enhance clinical relevance and support more effective information retrieval and prediction from free-text EHR data.

Our findings in this study are practically and clinically important for improving malnutrition management among older adults in RAC. The methodology presented in this work can also be applied by other researchers or practitioners to develop malnutrition prediction models using similar approaches. Furthermore, our study highlights the potential integration of the domain-adapted LLM as a clinical decision support tool rather than a standalone diagnostic system. We emphasise that our contribution lies in the development of the methodology, which demonstrates how such prediction models could be used to flag residents at potential risk of malnutrition through automated screening of their EHR data. This approach has the potential to assist nurses and clinicians in efficiently identifying at-risk individuals and implementing timely, tailored prevention and intervention strategies. In addition, this study demonstrates the feasibility of using a robust, domain-adapted LLM, such as RoBERTa, which can be further leveraged by researchers to develop optimal models for various downstream clinical tasks, supporting evidence-based practice and enhancing the quality of care in aged care settings.

The area of LLMs is evolving at a rapid pace; recently, decoding models such as GPT-3.5 and GPT-4, although widely doubted, have revolutionised the whole field of NLP. However, there remains a paucity of usable models for healthcare systems [24]. We intend to compare the prediction ability of our encoder-based LLM with one of the more recent and advanced decoder-based LLMs, such as Llama 2 [28]. We will also gather additional nursing notes to enhance the model’s performance, particularly for the second task.

### Limitations and Future Work

This study has several notable limitations. Firstly, large language models typically require substantial training data to achieve high predictive accuracy [15]. Although our models were trained on a relatively large dataset of nursing progress notes from 40 RACFs, the dataset size for the malnutrition prediction task remains modest, which may have impacted model performance. Expanding the dataset in future work could improve predictive accuracy and produce more robust models.

Secondly, while the included RACFs represent a diverse range of facilities within a single organisation, all sites share similar policies and electronic data collection practices. This limits the generalizability of the models to RACFs with different operational strategies or documentation practices. Additionally, nursing notes may contain inherent biases due to variations in documentation styles, subjective observations, or incomplete reporting, which could influence model predictions. Future research should focus on cross-institutional validation to ensure broader applicability across diverse RAC settings. Moreover, integrating multimodal data sources, such as laboratory results, dietary intake records, and vital signs, could enhance predictive accuracy and clinical relevance. Incorporating explainability methods, such as SHAP or attention visualisation, would also improve transparency and interpretability, supporting the integration of such models into clinical decision-making workflows.

Thirdly, this study did not include human validation of the model’s outputs. The absence of clinical validation limits the assessment of practical applicability, as end-user acceptance and trust are critical for successful deployment in healthcare settings. In future work, we plan to collaborate with healthcare professionals, including nurses and dietitians, to validate the model’s predictions, assess agreement with clinical judgement, and explore how such tools can be effectively integrated into existing aged care workflows.

Fourthly, while the model successfully extracted 46 malnutrition risk factors from nursing progress notes, we did not analyse the relative contribution or importance of each factor to the model’s predictions. This limits understanding of how the model prioritises different clinical indicators and reduces interpretability from a clinical perspective. In future work, we aim to address this by conducting a feature importance or attention-based explainability analysis to identify the most influential predictors. Such analysis would enhance transparency and support healthcare professionals in interpreting and applying model outcomes for informed clinical decision-making.

Fifthly, this study did not include a detailed error or failure case analysis to identify situations where the model produced incorrect predictions. Understanding these failure cases is essential for evaluating model reliability, detecting potential biases, and improving robustness in clinical applications. We acknowledge this as a limitation and plan to address it in future work by systematically examining misclassified cases and identifying underlying causes of prediction errors. This analysis will help refine the model, enhance its performance, and establish clearer boundaries for appropriate use in healthcare decision-making.

Finally, our study did not perform ablation experiments comparing different integration or fusion methods, which could potentially enhance model performance and interpretability. Future work will explore advanced architectures, such as attention-based fusion, cross-modal transformers, and hierarchical combination strategies.

## 5. Conclusions

To address the critical issue of malnutrition in older adults, our study presents three main contributions: (1) we demonstrated an encoder-based RAC domain-specific LLM by fine-tuning the foundational RoBERTa model on nursing progress notes from RACFs; (2) we showed how the resulting embeddings can be effectively integrated for two downstream clinical NLP tasks, malnutrition note identification and malnutrition prediction; and (3) we developed a method to handle long nursing notes by truncating and aggregating text into segments that fit within RoBERTa’s 512-token limit, enabling the use of extended clinical notes. The fine-tuned RAC domain-specific LLM achieved strong performance in both tasks, emphasising that domain-specific fine-tuning enhances the predictive capability of foundational LLMs. Additionally, we demonstrated that combining structured malnutrition risk factors with free-text embeddings further improves model performance, offering a practical approach for more accurate clinical predictions. Overall, our findings provide a comprehensive methodology for fine-tuning and integrating domain-adapted LLMs in clinical prediction tasks, which can be extended to other applications in aged care and broader healthcare settings.

## Figures and Tables

**Table 1:** The proportion of malnourished clients in the studied population (*n* = 4405).

	Well-Nourished (*n* = 3204)	Malnourished (*n* = 1201)
Age	Mean (SD)	Mean (SD)
	85.2 (8.9)	85.1 (8.9)
Female	2071 (74%)	726 (26%)
Male	1133 (70%)	475 (30%)

## Data Availability

Data is not available to comply with the Australian laws on privacy protection.

## Supplementary Materials

The following supporting information can be downloaded at https://www.mdpi.com/article/10.3390/healthcare13202614/s1, Figure S1: The RAC domain-specific LLM training, (A) validation loss, (B) train loss; Figure S2: The Malnutrition prediction model; Figure S3: AUPRC (A) and AUROC (B) of malnutrition note identification model—BioClinicalBERT; Figure S4: AUPRC (A) and AUROC (B) of malnutrition note identification model—RoBERTa base; Figure S5: AUPRC (A) and AUROC (B) of malnutrition note identification model—RAC domain-specific LLM; Figure S6: AUPRC (A) and AUROC (B) for malnutrition prediction model—BioClinicalBERT; Figure S7: AUPRC (A) and AUROC (B) for malnutrition prediction model—RoBERTa base; Figure S8: AUPRC (A) and AUROC (B) for malnutrition prediction model—RAC domain-specific LLM; Figure S9: AUPRC (A) and AUROC (B) for malnutrition prediction model—RAC domain-specific LLM with risk factor layer; Table S1: Example of tokenization and 15% whole word masking; Table S2: Performance of the five machine learning models on the task of identifying malnutrition notes; Table S3: Results of the malnutrition prediction model.
