# Supplementary material for: Fine-Tuning Large Language Models for Effective Nutrition Support in Residential Aged Care: A Domain Expertise Approach
Source: Healthcare (Basel). 2025 Oct 17;13(20):2614. doi: 10.3390/healthcare13202614 (PMC12564718; doi:10.3390/healthcare13202614)
Supplement: Supplementary file 1 [file healthcare-13-02614-s001.zip › healthcare-3836233-supplementary.pdf]

Fine-Tuning Large Language Models for Effective Nutrition Support in Residential Aged Care: A Domain Expertise Approach

Authors:

Mohammad Alkhalaf [1], Dinithi Vithanage [2], Jun Shen [2], Hui Chen (Rita) Chang [3], Chao Deng [4] and Ping Yu [2,*]

1 School of Computer Science, Qassim University, Qassim 51452, Saudi Arabia; mohklf@gmail.com

2 Centre for Digital Transformation, School of Computing and Information Technology, Faculty of Engineering and Information Sciences, University of Wollongong, Wollongong, NSW 2522, Australia; dsv912@uowmail.edu.au (D.V.); jshen@uow.edu.au (J.S.)

3 School of Nursing and Midwifery, Western Sydney University, Penrith, NSW 2751, Australia; rita.chang@westernsydney.edu.au

4 School of Medical, Indigenous and Health Sciences, University of Wollongong, Wollongong, NSW 2522, Australia; chao@uow.edu.au

*Correspondence Author:

Ping Yu,
Director, Centre for Digital Transformation
School of Computing and Information Technology.
Faculty of Engineering and Information Sciences
Northfield Ave, University of Wollongong, Wollongong, NSW 2522, Australia
Email: ping@uow.edu.au
Phone: +61-2-42215412

Supplementary Table S1: Example of tokenization and 15% whole word masking

Original	Client had physical impairment and unsteady gait related to Parkinson disease and has high malnutrition risk.
Tokenized	'ĠClient', 'Ġhad', 'Ġphysical', 'Ġimpairment', 'Ġand', 'Ġunst', 'ead', 'y', 'Ġg', 'ait', 'Ġrelated', 'Ġto', 'ĠParkinson', 'Ġdisease', 'Ġand', 'Ġhas', 'Ġhigh', 'Ġmalnutrition', 'Ġrisk', '.'
Masking (Whole word masking is in red colour)	Client had physical impairment<mask><mask><mask> gait<mask> to Parkinson disease and has high malnutrition <mask>.

A

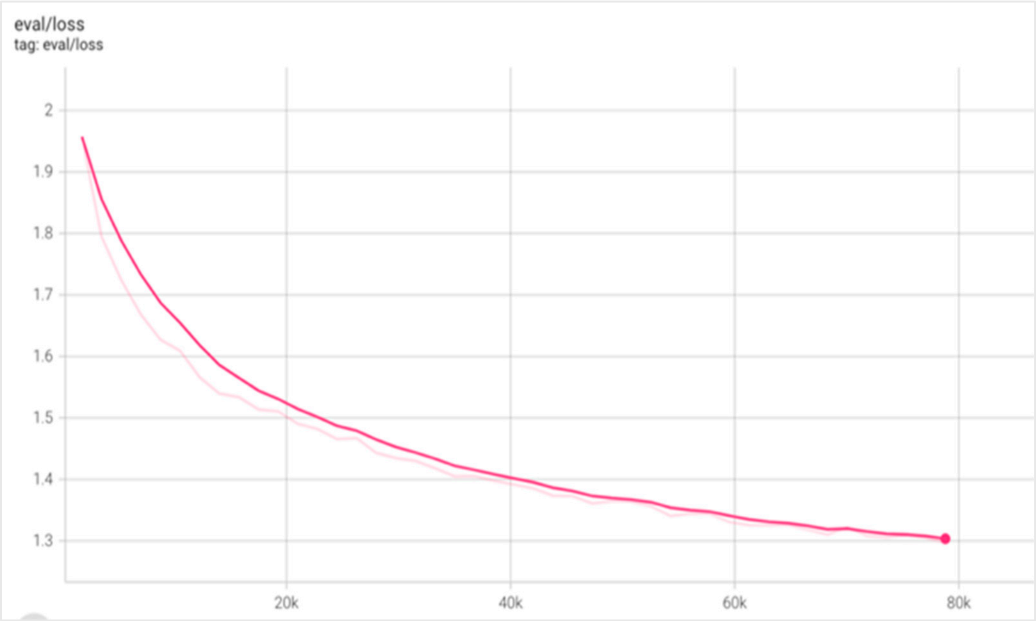

B

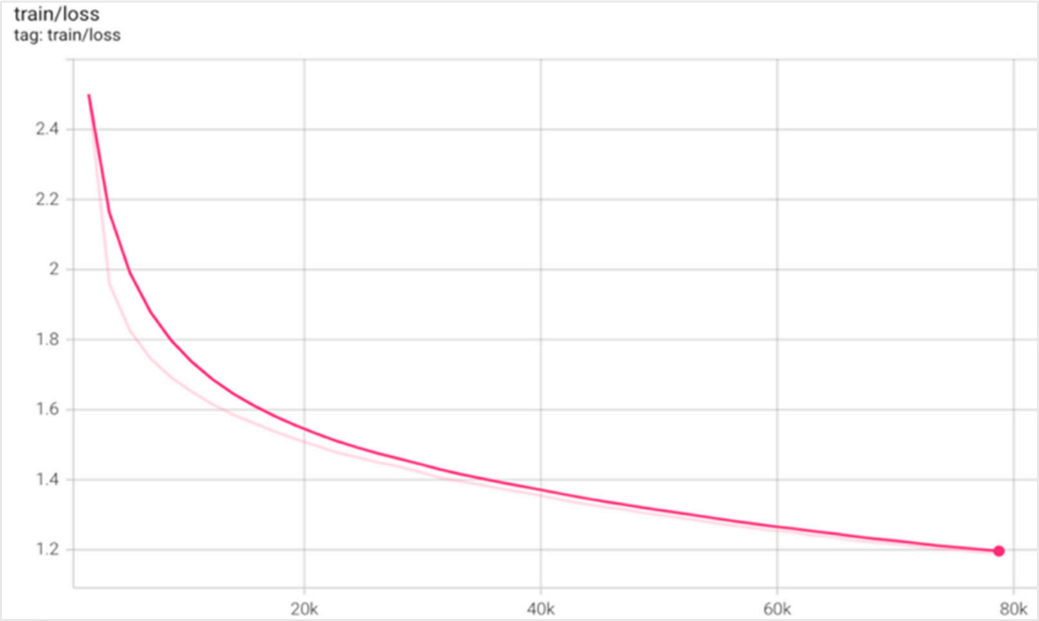

Supplementary Figure S1. The RAC domain-specific LLM training, (A) validation loss, (B) train loss

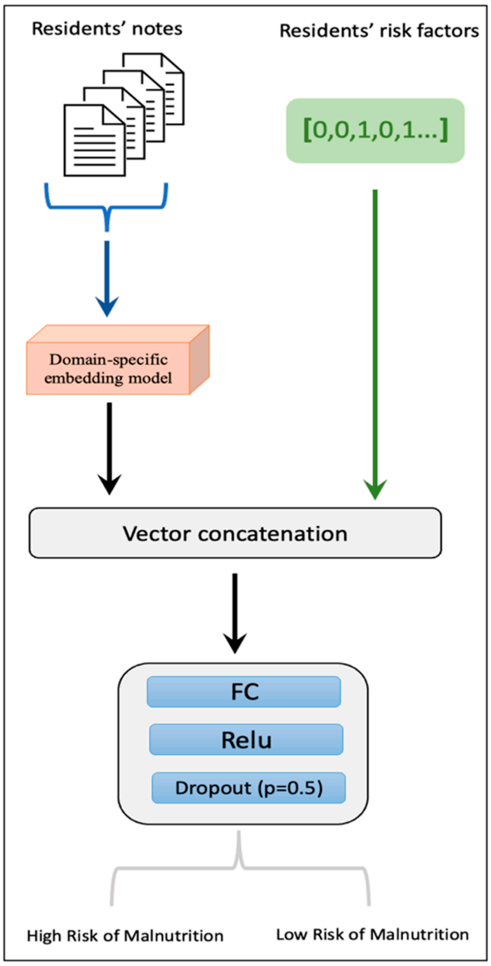

FC: Fully connected layer
P: Probability

Supplementary Figure S2. The Malnutrition prediction model

A

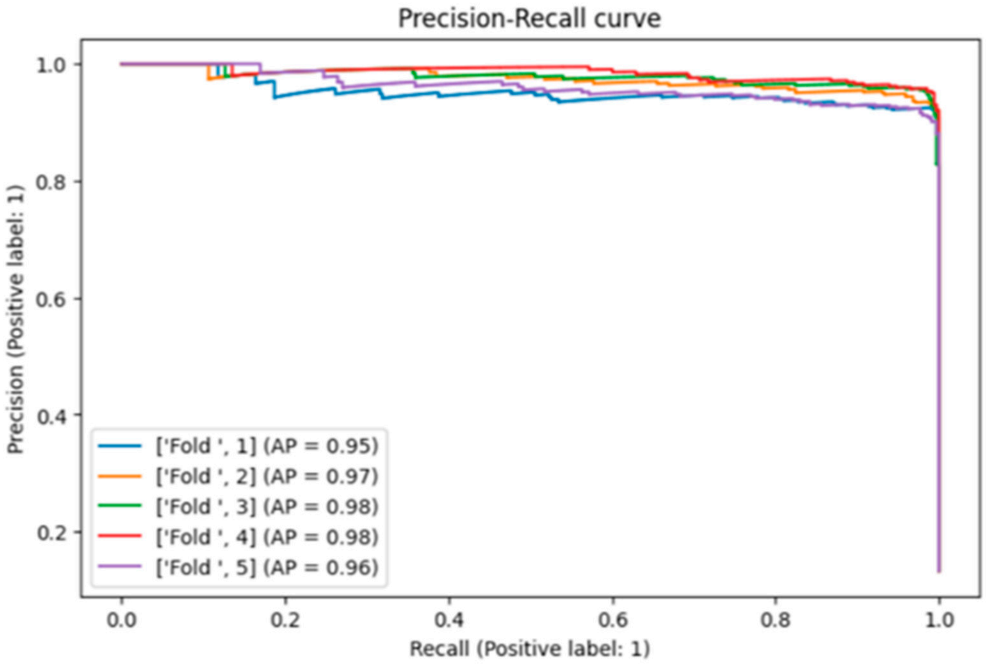

B

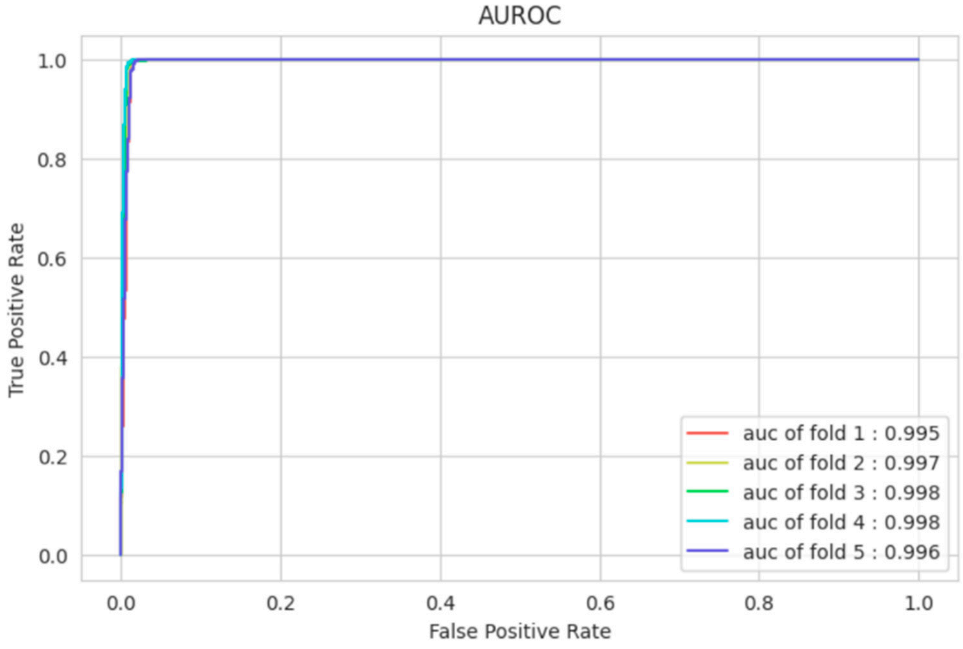

Supplementary Figure S3. AUPRC (A) and AUROC (B) of malnutrition note identification model -BioClinicalBERT

A

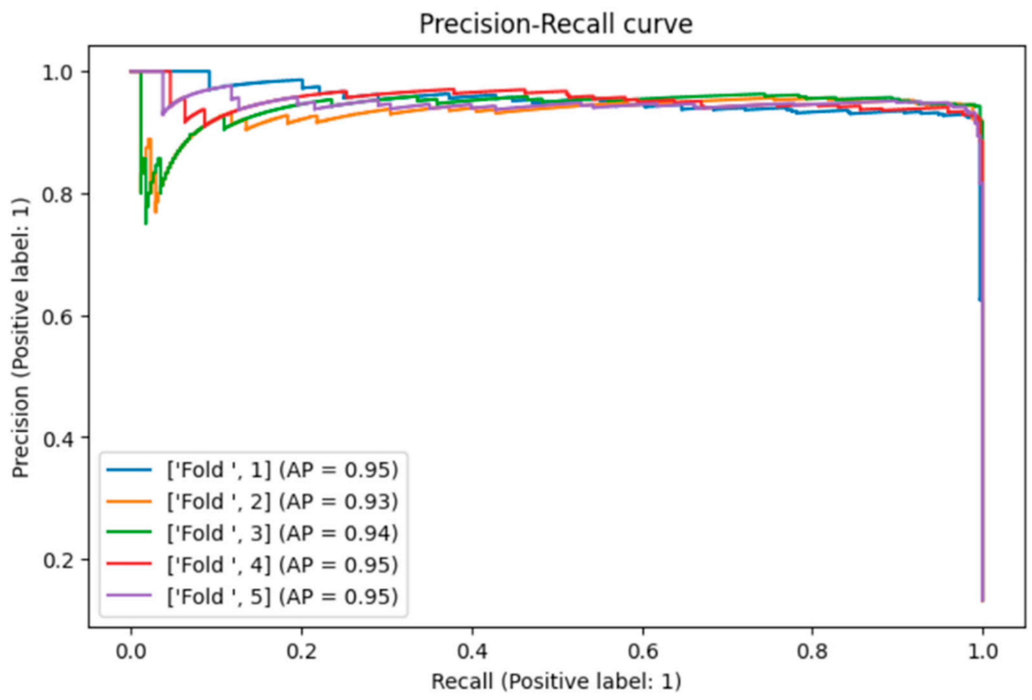

B

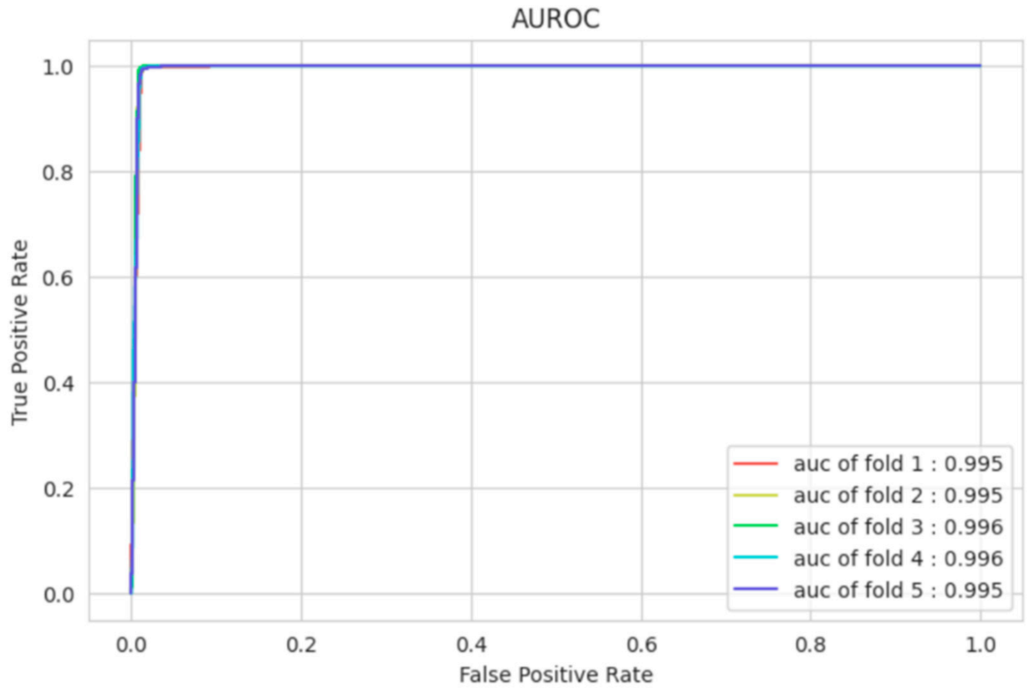

Supplementary Figure S4. AUPRC (A) and AUROC (B) of malnutrition note identification model – RoBERTa base

A

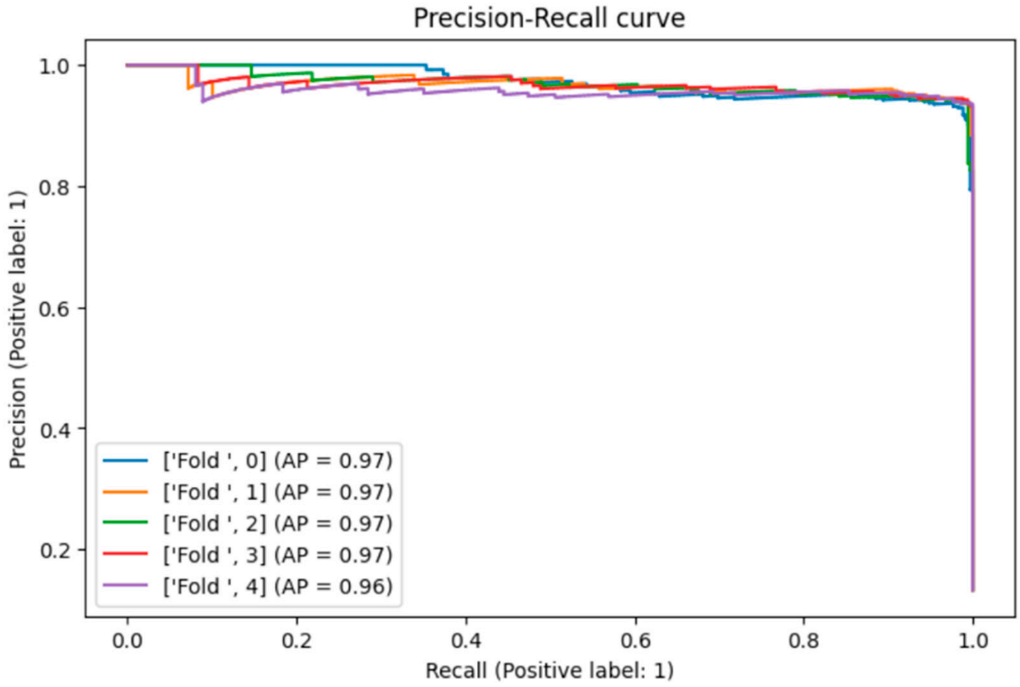

B

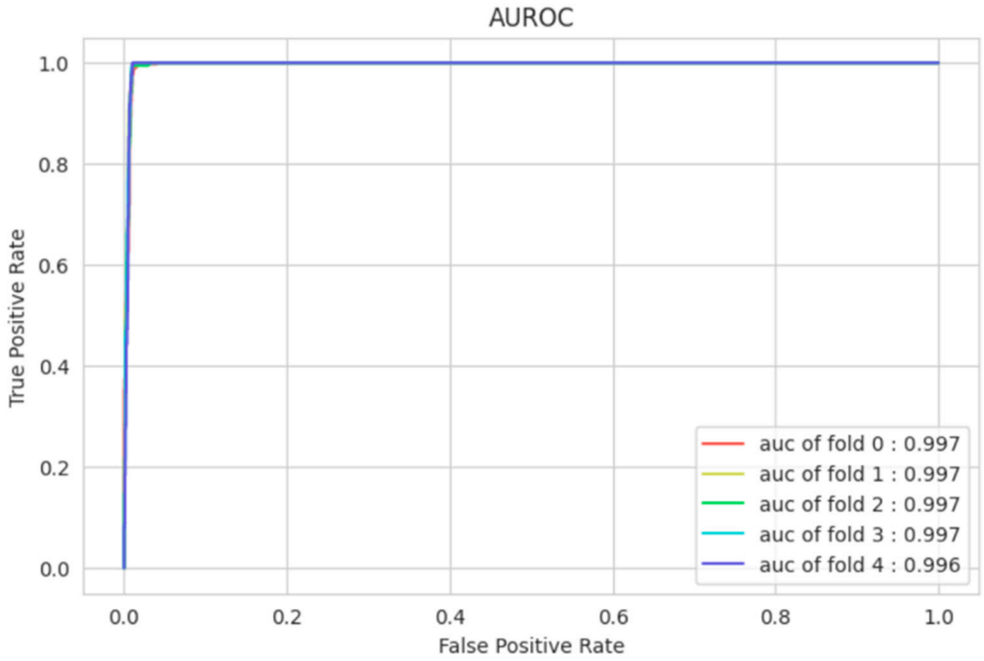

Supplementary Figure S5. AUPRC (A) and AUROC (B) of malnutrition note identification model - RAC domain-specific LLM

A

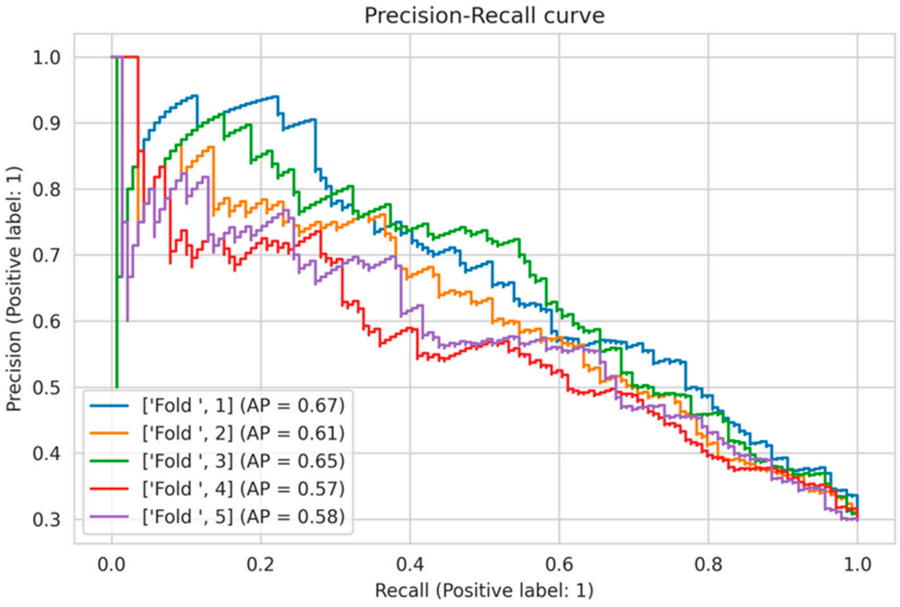

B

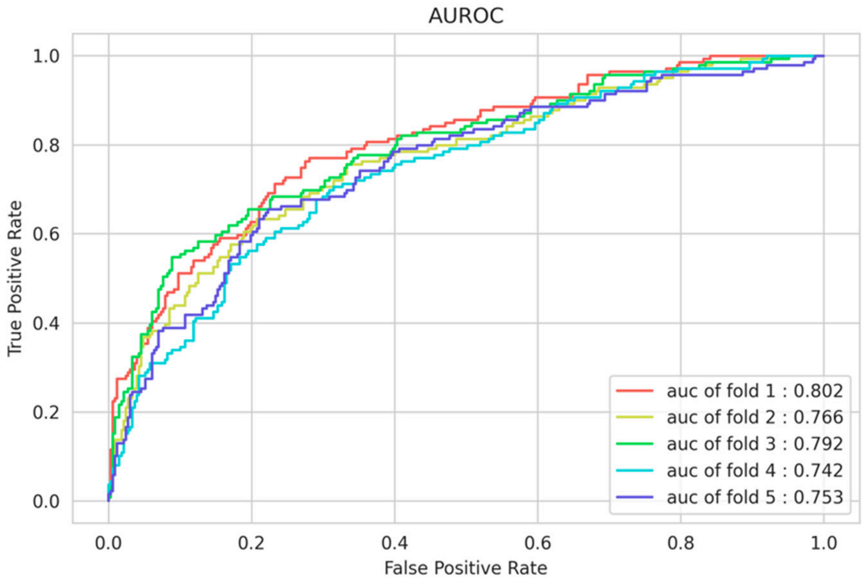

Supplementary Figure S6. AUPRC (A) and AUROC (B) for malnutrition prediction model - BioClinicalBERT

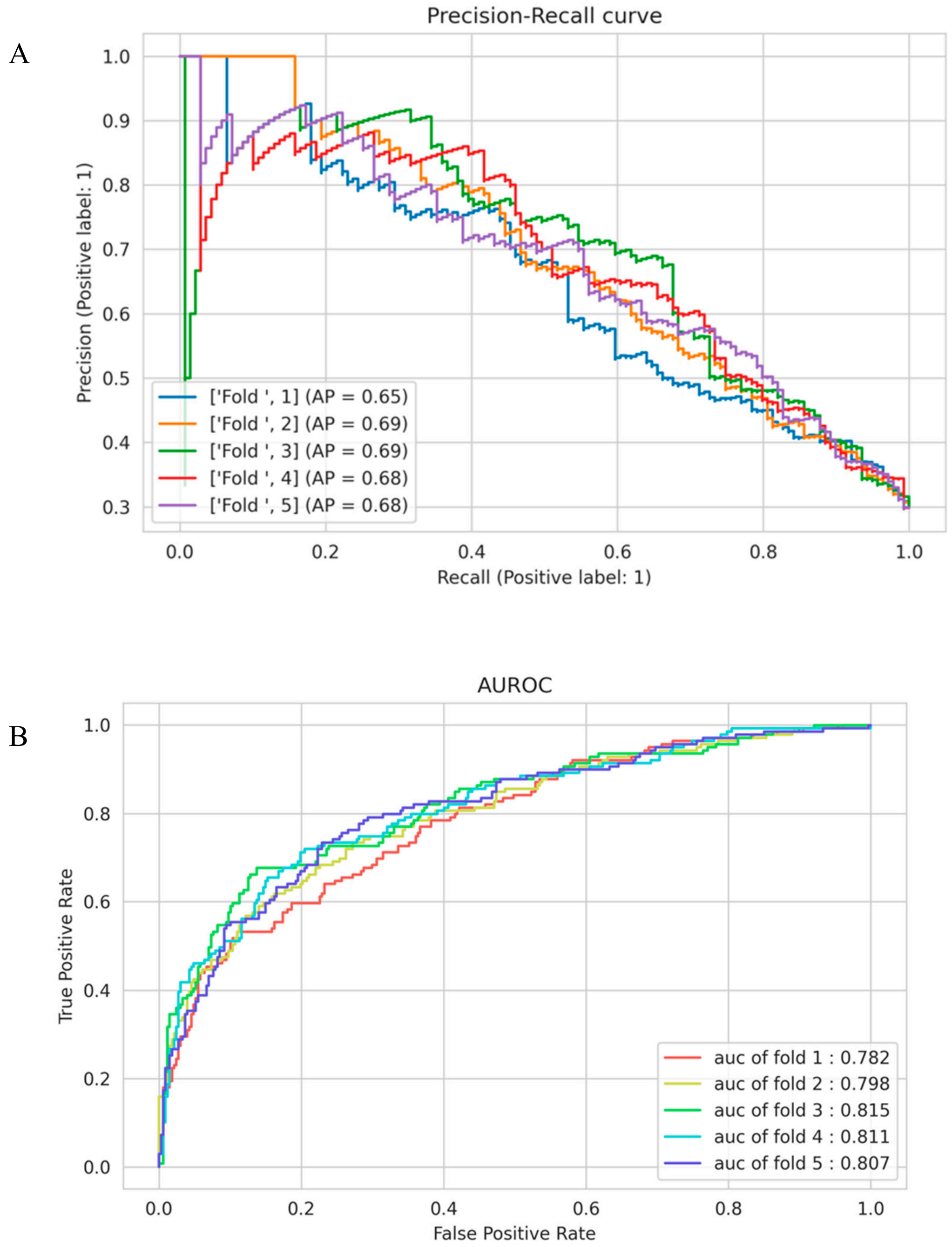

Supplementary Figure S7. AUPRC (A) and AUROC (B) for malnutrition prediction model - RoBERTa base

A

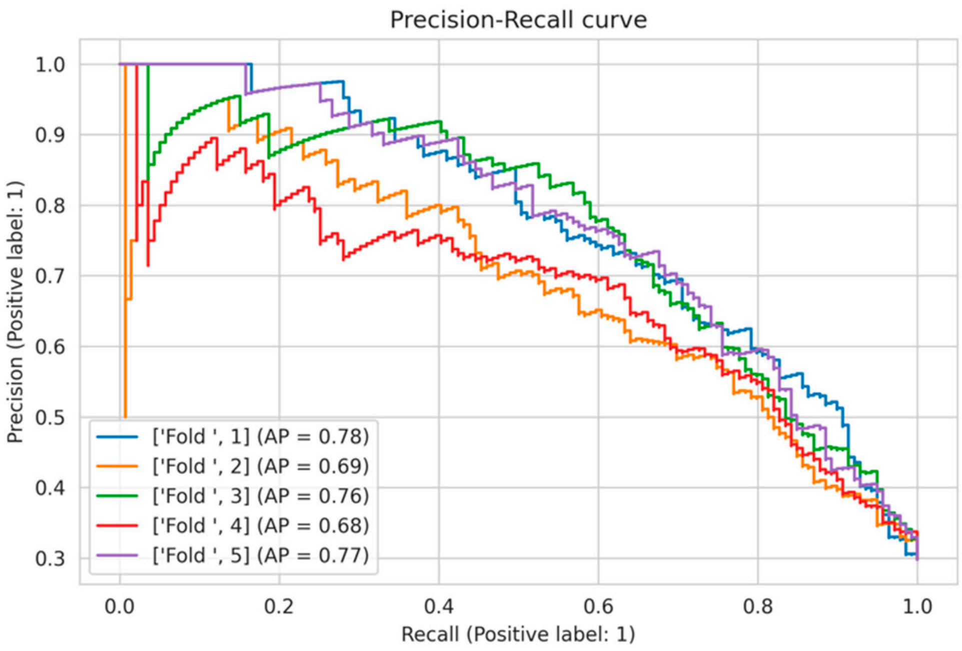

B

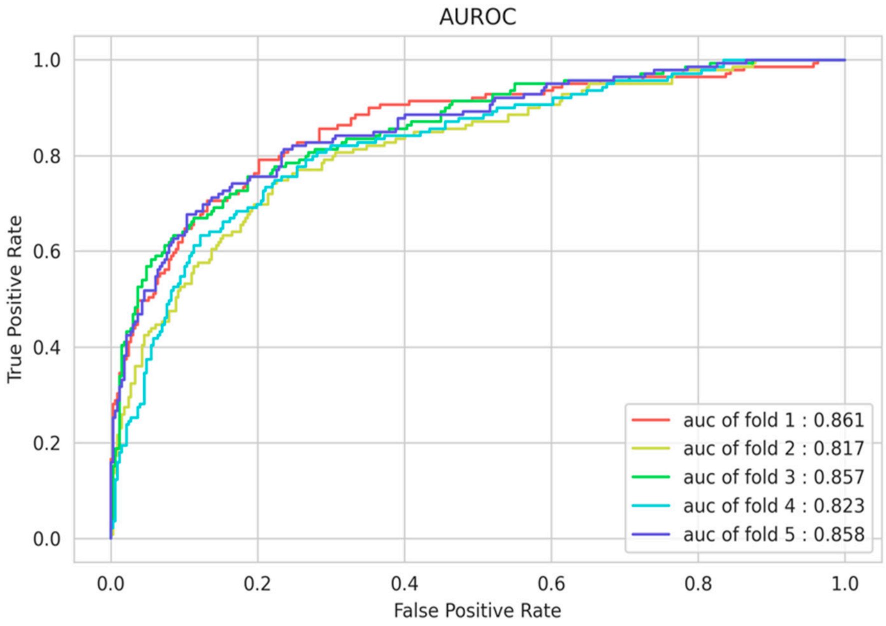

Supplementary Figure S8. AUPRC (A) and AUROC (B) for malnutrition prediction model - RAC domain-specific LLM

A

B

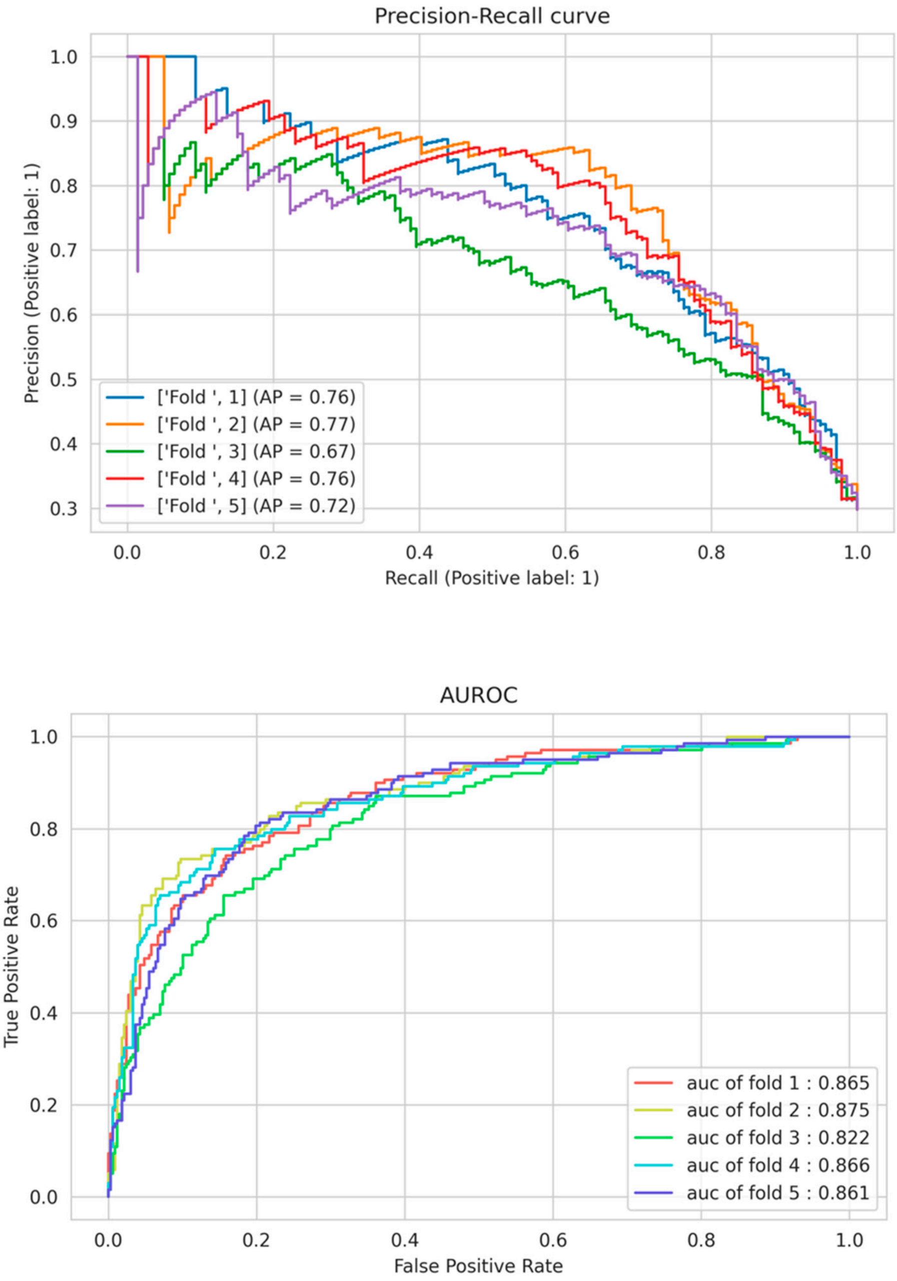

Supplementary Figure S9. AUPRC (A) and AUROC (B) for malnutrition prediction model - RAC domain-specific LLM with risk factor layer

Supplementary Table S2: Performance of the five machine learning models on the task of identifying malnutrition notes

Model	Precision (95% CI)	Recall (95% CI)	F1-Score* (95% CI)	Specificity (95% CI)	AUPRC* (95% CI)	AUROC* (95% CI)
BOW+TF-IDF	0.903 (0.90-0.91)	0.842 (0.83 – 0.85)	0.871 (0.87 – 0.88)	0.986 (0.98 – 0.99)	0.883 (0.88 – 0.89)	0.914 (0.91 – 0.92)
GloVe (6B, 300d)	0.855 (0.80 – 0.91)	0.959 (0.94 – 0.98)	0.903 (0.88 – 0.93)	0.977 (0.97 – 0.99)	0.931 (0.90 – 0.96)	0.992 (0.99 - 1.0)
BlueBERT	0.898 (0.89 – 0.90)	0.962 (0.94 – 0.97)	0.951 (0.91 – 0.94)	0.980 (0.97 – 0.99)	0.942 (0.93 – 0.95)	**1.0 (1.0 – 1.0)**
ClinicalBERT	0.901 (0.90 – 0.92)	0.960 (0.96 – 0.97)	0.960 (0.96 – 0.97)	**0.990 (0.99 – 0.99)**	0.952 (0.94 – 0.96)	**1.0 (1.0 – 1.0)**
BioClinicalBERT	0.932 (0.91 – 0.95)	0.970 (0.98 – 0.99)	0.962 (0.95 – 0.97)	**0.990 (0.99 – 0.99)**	0.966 (0.95 – 0.98)	**1.0 (1.0 – 1.0)**
PubBERT	0.935 (0.92 – 0.95)	0.980 (0.98 – 0.99)	0.962 (0.95 – 0.97)	**0.990 (0.99 – 0.99)**	0.966 (0.95 – 0.98)	**1.0 (1.0 – 1.0)**
roberta-base	0.936 (0.92 – 0.95)	**0.994 (0.99 – 1.0)**	0.964 (0.96 – 0.97)	**0.990 (0.99 – 0.99)**	0.958 (0.94 – 0.97)	**1.0 (1.0 – 1.0)**
RAC domain-specific LLM	**0.942 (0.94 – 0.95)**	**0.994 (0.99 – 1.0)**	**0.966 (0.96 – 0.97)**	**0.990 (0.99 – 0.99)**	**0.978 (0.97 – 0.99)**	**1.0 (1.0 – 1.0)**

*F1-score computed using 0.5 threshold
* AUPRC and AUCROC computed across various threshold values

Supplementary Table S3: Results of the malnutrition prediction model

Model	Precision (95% CI)	Recall (95% CI)	F1-Score* (95% CI)	Specificity (95% CI)	AUPRC* (95% CI)	AUROC* (95% CI)
BOW+ TF-IDF	**0.658 (0.63 – 0.69)**	0.483 (0.45 – 0.51)	0.557 (0.53 – 0.58)	**0.872 (0.86 – 0.89)**	0.658 (0.64 – 0.68)	0.677 (0.66 – 0.69)
GloVe (6B, 300d)	0.450 (0.43 – 0.47)	0.375 (0.19 – 0.56)	0.396 (0.28 – 0.51)	0.766 (0.66 – 0.87)	0.412 (0.40 – 0.43)	0.609 (0.59 – 0.63)
BlueBERT	0.481 (0.43 – 0.47)	0.389 (0.45 – 0.56)	0.421 (0.46 – 0.52)	0.768 (0.70 – 0.80)	0.521 (0.45 – 0.47)	0.671 (0.64 – 0.68)
ClinicalBERT	0.550 (0.50 – 0.58)	0.601 (0.55 – 0.65)	0.550 (0.52 – 0.56)	0.752 (0.74 – 0.78)	0.603 (0.58 – 0.66)	0.771 (0.74 – 0.80)
BioClinicalBERT	0.554 (0.51 – 0.59)	0.617 (0.54 – 0.70)	0.582 (0.54 – 0.62)	0.787 (0.74 – 0.84)	0.613 (0.56 – 0.67)	0.771 (0.74 – 0.80)
PubBERT	0.550 (0.52 – 0.56)	0.651 (0.59 – 0.72)	0.600 (0.58 – 0.63)	0.772 (0.74 – 0.83)	0.600 (0.56 – 0.65)	0.750 (0.74 – 0.80)
roberta-base	0.579 (0.50 – 0.66)	0.662 (0.59 – 0.74)	0.614 (0.58 – 0.65)	0.789 (0.71 – 0.87)	0.677 (0.66 – 0.70)	0.803 (0.79 – 0.82)
RAC domain-specific LLM	0.592 (0.50 – 0.68)	0.751 (0.64 – 0.86)	0.655 (0.62 – 0.69)	0.766 (0.63 – 0.90)	0.734 (0.67 – 0.80)	0.843 (0.82 – 0.87)
RAC domain-specific LLM + risk factors	0.655 (0.53 – 0.70)	**0.790 (0.71 – 0.87)**	**0.687 (0.65 – 0.72)**	0.780 (0.67 – 0.89)	**0.735 (0.68 – 0.79)**	**0.858 (0.83 – 0.88)**

*F1-score computed using 0.5 threshold
* AUPRC and AUCROC computed across various threshold values
